# Whisker and Nose Tactile Sense Guide Rat Behavior in a Skilled Reaching Task

**DOI:** 10.3389/fnbeh.2018.00024

**Published:** 2018-02-21

**Authors:** Pierantonio Parmiani, Cristina Lucchetti, Gianfranco Franchi

**Affiliations:** ^1^Department of Biomedical and Specialty Surgical Sciences, Section of Human Physiology, University of Ferrara, Ferrara, Italy; ^2^Center for Translational Neurophysiology, Istituto Italiano di Tecnologia, Ferrara, Italy; ^3^Department of Biomedical, Metabolic and Neural Sciences, Section of Physiology and Neuroscience, University of Modena and Reggio Emilia, Modena, Italy

**Keywords:** whisker sense, skilled reaching, whisker trimming, ION severing, rat

## Abstract

Skilled reaching is a complex movement in which a forelimb is extended to grasp food for eating. Video-recordings analysis of control rats enables us to distinguish several components of skilled reaching: Orient, approaching the front wall of the reaching box and poking the nose into the slot to locate the food pellet; Transport, advancing the forelimb through the slot to reach-grasp the pellet; and Withdrawal of the grasped food to eat. Although food location and skilled reaching is guided by olfaction, the importance of whisker/nose tactile sense in rats suggests that this too could play a role in reaching behavior. To test this hypothesis, we studied skilled reaching in rats trained in a single-pellet reaching task before and after bilateral whisker trimming and bilateral infraorbital nerve (ION) severing. During the task, bilaterally trimmed rats showed impaired Orient with respect to controls. Specifically, they detected the presence of the wall by hitting it with their nose (rather than their whiskers), and then located the slot through repetitive nose touches. The number of nose touches preceding poking was significantly higher in comparison to controls. On the other hand, macrovibrissae trimming resulted in no change in reaching/grasping or withdrawal components of skilled reaching. Bilaterally ION-severed rats, displayed a marked change in the structure of their skilled reaching. With respect to controls, in ION-severed rats: (a) approaches to the front wall were significantly reduced at 3–5 and 6–8 days; (b) nose pokes were significantly reduced at 3–5 days, and the slot was only located after many repetitive nose touches; (c) the reaching-grasping-retracting movement never appeared at 3–5 days; (d) explorative paw movements, equal to zero in controls, reached significance at 9–11 days; and (e) the restored reaching-grasping-retracting sequence was globally slower than in controls, but the success rate was the same. These findings strongly indicate that whisker trimming affected Orient, but not the reaching-grasping movement, while ION severing impaired both Orient (persistently) and reaching-grasping-retracting (transiently, for 1–2 weeks) components of skilled reaching in rats.

## Introduction

Sensory-motor integration involves coupling the sensory system and motor system. It is not a static process, since for a given behavior there is no one single sensory input and no one single motor command. Neural responses at almost every stage of a sensorimotor pathway can be modified at short and long timescales by biophysical and synaptic processes, recurrent and feedback connections, and learning, as well as many other internal and external variables (Huston and Jayaraman, 2011; Sereno and Huang, 2014; Luo et al., 2017). Besides this, multisensory integration allows information from the different sensory modalities, such as sight, sound, touch, taste, smell and self-motion, to guide motor behavior (Kleinfeld and Deschênes, 2011; Kleinfeld et al., 2014; Miller et al., 2017).

A suitable model for studying multisensory integration in motor behavior is the so called “skilled reaching”, a complex movement common to many animal species, in which a forelimb is extended to grasp food that is placed in the mouth for eating. Multiple parallel parietofrontal circuits, devoted to specific sensorimotor transformations during skilled reaching, have been described in monkeys (Matelli and Luppino, 2001; Omrani et al., 2016, 2017), and evidence from functional brain imaging studies suggests that the organization of this complex behavior is based on the same principles in humans (Sacrey et al., 2009; Filimon, 2010).

For many years, rodents have been trained in skilled reaching in order to study diverse aspects of this behavior, such as neural control of the forelimb (Alaverdashvili et al., 2008; Kawai et al., 2015), functional recovery from neural injury (Moon et al., 2009), and assessment for brain damage (Klein et al., 2012; Alaverdashvili and Whishaw, 2013). Indeed, the skilled reaching task is a composite behavior involving a sequence of movements, namely: orienting of the rat within the reaching box, food localization, reaching-grasping food, and bringing food to the mouth (Alaverdashvili et al., 2008). The observation that skilled reaching-grasping is relatively inflexible supports the notion that it is produced by complex fixed neural circuitry (Metz and Whishaw, 2000). It is probable that the intrinsic recurrent synaptic connections between sensory and motor cortices and within the motor cortex are the cortical circuits that allow coupling between the spatial location of the pellet and the spatial location to which the limb is commanded to move (Capaday et al., 2013; Feldmeyer et al., 2013; Stüttgen and Schwarz, 2018). The projection from the vibrissa-S1 (vS1) is most likely the main source of sensory input to the vibrissa-M1 (vM1; Farkas et al., 1999; Chakrabarti et al., 2008; Aronoff et al., 2010; Hooks, 2016); specifically the region TZ receives S1 input, while the medial of vM1 areas do not (Smith and Alloway, 2013; Schwarz and Chakrabarti, 2015). The horizontal reciprocal cortico-cortical connections between vM1 and forelimb-M1 (fM1; Huntley, 1997) could be the network directly involved in whisker, head and forelimb movement coordination during skilled reaching.

Interestingly, it has been shown that in the rat it is olfaction rather than vision that is used to locate food and guide reaching (Whishaw and Tomie, 1989; Hermer-Vazquez et al., 2007). Nevertheless, the relevance of whisker/nose sense in the rat (Brecht et al., 1997; Diamond and Arabzadeh, 2013; Kleinfeld et al., 2014), suggests that such information could also have a role in driving skilled reaching behavior. However, the effect of trigeminal input removal, i.e., sensory whisker pad and nose denervation, on skilled reaching performance has never before been evaluated, and it would be useful to determine which movement of the skilled reaching sequence is affected by whisker/nose sense suppression. To this end, we investigated orienting-reaching-grasping behavior in rats trained in a single-pellet reaching task, before and after infraorbital nerve (ION) severing and also after whisker trimming.

## Materials and Methods

### Subjects and Ethical Approval

Eleven adult male albino Wistar rats, each weighing between 280 g and 330 g, raised in the University of Ferrara animal house, were used for this study. In five rats, the ION of both sides was cut (bilaterally ION-severed rats); in three rats the macrovibrissae were bilaterally trimmed (bilaterally trimmed rats); and in three rats a sham surgery without ION severing was performed bilaterally. The experimental plan was designed in compliance with Italian law regarding the care and use of experimental animals (DL26/14), and approved by both the University of Ferrara Ethics Board (OBA) and the Italian Ministry of Health; and all procedures complied with the ethical standards of the European Council Directive of 4 March 2014 regarding the treatment of animals in research.

### Feeding and Food Restriction

Rats were housed in polycarbonate cages (53 cm long, 37 cm wide, and 21 cm deep) with sawdust bedding, in groups of three or four in a colony, under a 12 h:12 h light/dark cycle with light starting at 07:30 h. All testing and training was performed during the light phase of the cycle at the same time of day. The animals received water *ad libitum*, but were food-deprived before the start of training. The week before training began, each rat received 20 banana-flavored round food pellets (Rodent Tab 45 mg, AIN-76A, TestDiet, Richmond, VA, USA) 1 h prior to the daily fodder ration. These pellets would later serve as reaching targets in a single-pellet reaching box. Each animal maintained about 90% of their initial body weight throughout the experiment; to maintain body weight, the rats were given an additional amount of food in their home cages at least 1 h after finishing the training or testing session.

### Reaching Box and Single-Pellet Training

The reaching box was made of clear Plexiglass (340 × 390 × 134 mm wide), and was similar to that described by Metz and Whishaw (2000) and Alaverdashvili et al. (2008). Briefly, the middle of the front wall featured a 10-mm-wide, vertical opening to allow the animal to reach for the pellets. These were placed on a shelf, 15 mm wide and 20 mm long, which was attached outside the front wall of the box, 25 mm above the base. The upper side of the shelf, aligned to the midline of the box, featured a round indentation (diameter 7 mm, depth 2.5 mm, distance from the front wall 10 mm) for food pellet positioning. During pre-training (about 1 week) the rat was placed in the box for 20-min daily sessions during which it was allowed to explore the reaching box and encounter the food pellets placed on the shelf to promote reaching-grasping through the slot. Pre-training ended when the rat started to reach with its forepaw for the food pellet. Training sessions also consisted of 20-min daily sessions during which the rat learned to grasp the pellet with the preferred paw. Paw preference was established when at least 60% of a minimum of 10 reach attempts were made using the left or the right forepaw. During training the rat was taught to advance from the posterior part of the box to the front wall, to sniff for the pellet on the shelf, and to perform the prehension sequence only if the pellet was present. If the pellet was absent, the rat was trained to go back to the posterior part of the box to start another trial. In order to facilitate learning of this movement sequence, a food pellet was dropped in the posterior part of the box in the first training sessions. Furthermore, at the end of each session several pellets were dropped into the box as a final reward, which gave us the chance to observe the rat’s spontaneous grasping from the floor under the different experimental conditions. It should be mentioned, however, that the experimental set up did not allow us to quantify this spontaneous behavior. The success level of prehension was scored in the last week of training and during recording sessions. The percentage success rate of each rat was calculated as the ratio between the prehension movements in which the rat brought a food pellet to its mouth and the total number of trials multiplied by 100. For each rat, training ended when the percentage success rate achieved almost 50% in the last consecutive sessions.

### Macrovibrissae Trimming

Each day of training began with a 5–10 min handling session in which the three rats were conditioned to tolerate being held firmly while its vibrissae were touched with a set of blunt-tipped scissors. This conditioning enabled us to cut off the vibrissae without anesthesia. After the rats achieve a pellet retrieval success rate above 50% in three consecutive daily sessions, all macrovibrissae—both mystacial and mental (whisker trident)—were bilaterally trimmed to <2 mm in length.

### Surgical Procedures: Infraorbital Nerve Severing

All surgical procedures were performed under ketamine anesthesia (80 mg kg^−1^ i.p., and then supplemental doses i.m. as needed). Under the operating microscope, the ION of both sides was exposed, separated from its adjacent tissues and ligated; it was then cut distally to eliminate all remaining fine branches. The proximal stump was dried and covered with acrylic tissue adhesive (Histoacryl) to prevent the proximal axons from sprouting. The skin was closed with 6–0 sutures, and then cleansed with an antibiotic solution. In the post-operative period, none of the five operated rats displayed complications such as self-mutilation, infection, or overt signs of discomfort. Clinical observation during natural whisking clearly showed that the deafferented whiskers displayed bilateral rhythmic movements, but did not suddenly retract when hit against targets, as would normally be the case. After deafferentation the whisker pad proved unreactive to mild pain-inducing sensory stimuli (i.e., light touching, squeezing or piercing). The loss of whisker pad sensitivity following deafferentation was clearly evident in all animals for the entire survival period. In the sham rats the ION was isolated from the surrounding tissues but left intact (for more details see Franchi, 2001).

### Histology

At the end of the experimental procedure, the animals were perfused transcardially. In each animal, gross post-perfusion examination of the injured nerves showed no nerve continuity at the acrylic stopper level. Under the operating microscope, care was taken to ensure that all ION fascicles had been tied and axotomized. In all animals studied, the exposed ION was cut proximal to the site of nerve injury and prepared for histological examination. After post-fixation in osmium tetroxide, toluidine blue was used to stain 1-μm thick sections. Morphological examination of sections (Axioskop Zeiss and DMC Polaroid camera for image acquisition) showed extensive degenerative processes involving all axons proximal to the lesion.

### Video-Recording of Rat Behavior

Throughout each experimental session, rats were video-recorded at 200 frames/s using a JVC GC-PX100 camera with a resolution of 640 × 360 pixels. The recording video camera was positioned so as to obtain a right or left lateral view of the animal inside the box, according to the handedness of the animal. Recorded videoclips were visualized off-line, and when necessary frame-by-frame analysis was performed using Avidemux 2.6 software^1^. Some rats used in these experiments were also used to carry out preliminary measures of skilled movement kinematics. This is the reason why, in some figures reported in this article, rat show markers on their head and forelimb, which, however, in no way interfered with the execution of movements.

In all rats, video-recordings were performed on three consecutive days before surgery. Then, whisker-trimmed rats were video-recorded daily from the day of trimming until the 17th day, whereas ION severed rats were video-recorded daily from the 3rd to the 17th day, as 3 days should be sufficient allow any depressive effect of the anesthetic to subside.

### Analysis of Rat Behavior

In the single-pellet reaching task, each rat is trained to approach the front wall of the reaching box from the rear, and to sniff through the slot to locate the pellet on the shelf. Then, if the pellet is present, the rat advances the preferred forelimb through the slot, grasps the pellet and then withdraws the paw to bring the pellet to the mouth for eating (Whishaw and Tomie, 1989). As the aim of our research was to define the role of whisker/nose tactile sensing in a single-pellet reaching task, we considered each trial as composed of three successive learned responses, which were analyzed separately: (1) Orient; (2) Transport; and (3) Withdrawal (Gharbawie and Whishaw, 2006; Alaverdashvili et al., 2008; Alaverdashvili and Whishaw, 2013). The Orient comprised the rat’s approach to the front wall, its locating the slot, and the nose poking through to sniff the pellet. The Transport involved the rat lifting its paw from the box floor and directing it through the slot to grasp the pellet. Withdrawal consisted of retracting the paw through the slot and placing the food pellet into the mouth for eating. These three components were chosen as they are present in all successfully completed trials, as missing one results in an error trial.

Qualitative and quantitative analysis, performed on video-recordings taken before and after bilateral whisker trimming and bilateral ION severing, provided insights into the structure of these learned responses. To study the temporal course of the effects of ION severing on behavior, the data were grouped into five successive 3-day intervals from the 3rd day after the lesion, and compared to data obtained in the same animal before the lesion. Data from each experimental group were pooled, and were presented as mean values with standard error. Under the different experimental conditions, reaching behavior was assessed by measures of total success. Specifically, a successful reach was defined as one in which an animal grasped a food pellet and placed it into the mouth. Total success was defined as: Success % = (number of pellets obtained/total number of reaching)*100.

**Figure 1:**
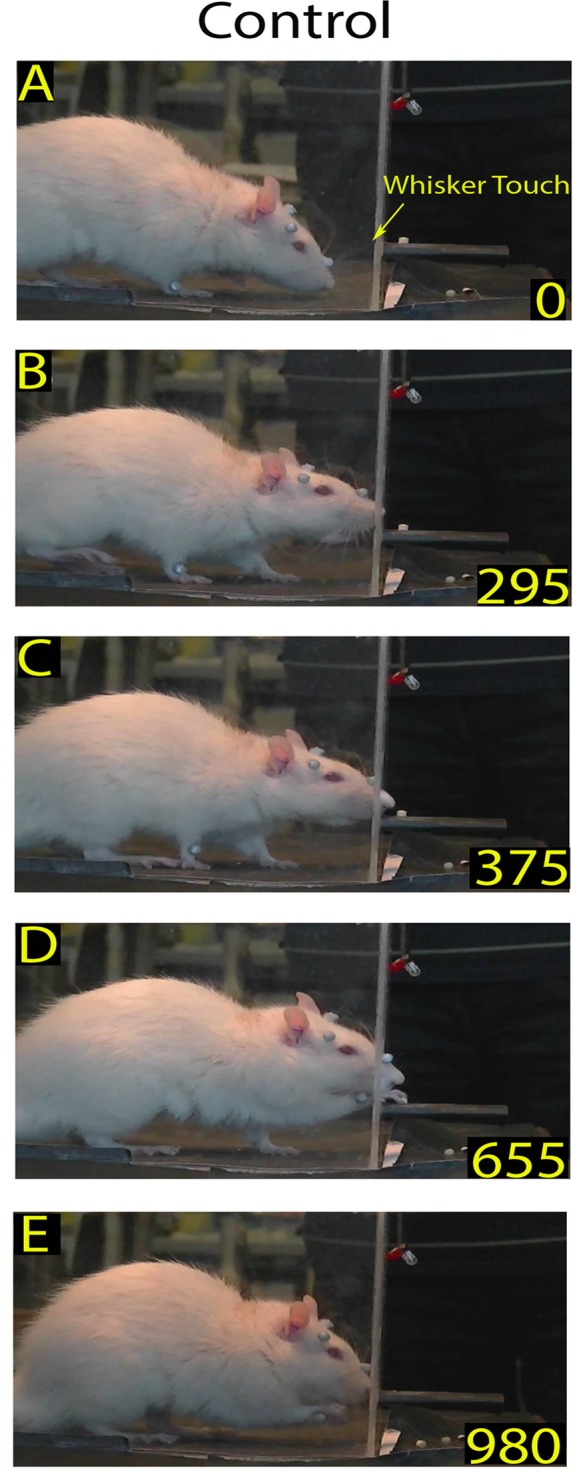
Example of a skilled reaching trial in control rat: video-recording from Rat 2. Each frame represents a salient step in the trial sequence. The top frame shows the first whiskers contact with the front wall during approaching before rat raised its head **(A)**; the bottom frame shows the rat putting the pellet into the mouth **(E)**; and the intermediate frames show the rat inserting its nose in the slot **(B)**, and during the reaching-grasping movement **(C,D)**, respectively. In each frame, the number at the bottom right is the timing (in ms). Markers are present (see “Materials and Methods” section).

**Figure 2:**
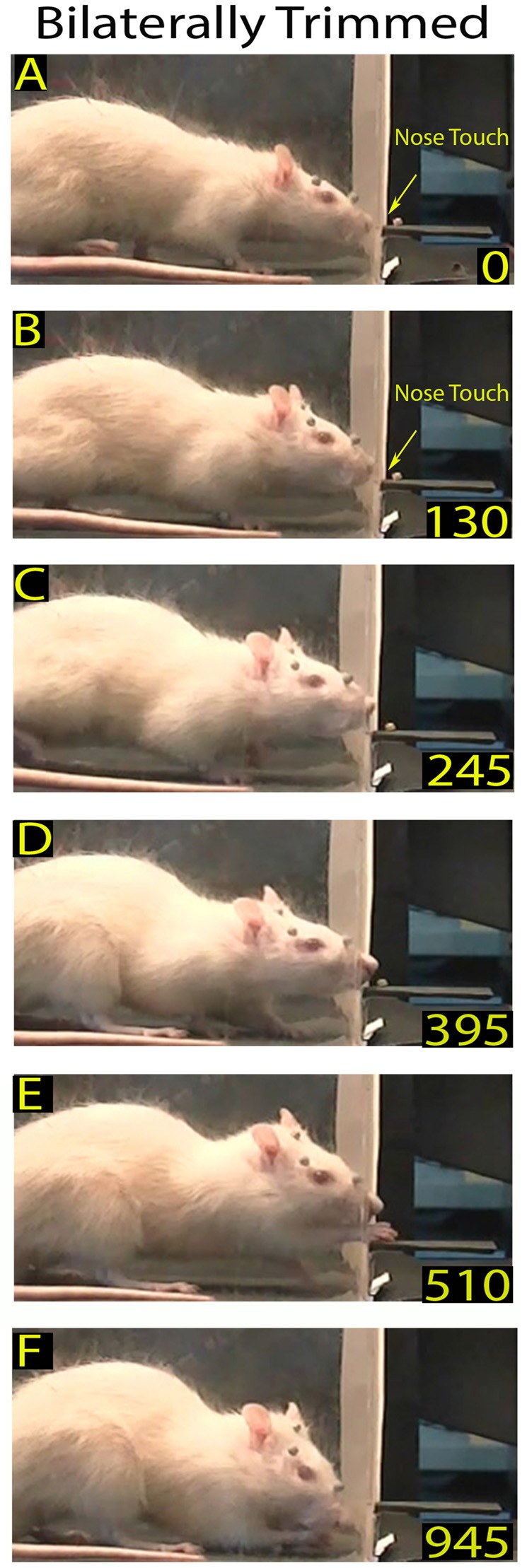
Example of a skilled reaching trial in a bilaterally trimmed rat. The top frames **(A,B)** show the first and second nose contacts with the front wall during approaching. Frames from **(C–F)** correspond to frames **(B–E)** of the Figure 1. Markers are present (see “Materials and Methods” section).

To analyze the Orient component of skilled reaching under the different experimental conditions, we first measured the frequency of each rat’s approaches toward the front wall. Then, to measure each rat’s ability to locate the slot, we calculated the percentage of nose poking with respect to the number of approaches. To measure the time spent in locating the slot, in control rats we identified the video frame in which the longer mystacial macrovibrissae first contacted the front wall of the box (Figure 1A) and the first frame showing the nose inside the slot (nose poke), and calculated the interval between. In trimmed and ION-severed rats, which explored the front wall by repetitive nose touches (Figures 2A,B), we calculated the number of nose touches before nose poking and the time between the first nose touch and nose poke (see Tables 1, 2).

**Table 1:** Behavioral parameters in control vs. trimmed rats.

	Control				Trimmed			
	R1	R3	R4	Group	R1	R3	R4	Group
Whisker-T Poke	285.75 * ± 17.00*	257.00 * ± 14.68*	191.05 * ± 11.77*	244.6 * ± 9.78*				
Nose-T Poke					196.25 * ± 15.01*	284.50 * ± 34.55*	104.10 * ± 12.14*	194.95 * ± 16.13*
N-Nose T	0.40 * ± 0.15*	0.85 * ± 0.08*	0.30 * ± 0.11*	0.52 * ± 0.07*	2.60 * ± 0.17*	2.85 * ± 0.31*	1.90 * ± 0.18*	2.45 * ± 0.14*
Poke-Start-Rch	93.10 * ± 8.72*	180.24 * ± 19.45*	173.57 * ± 17.03*	148.97 * ± 10.12*	124.29 * ± 13.63*	171.67 * ± 16.59*	177.48 * ± 16.12*	157.81 * ± 9.31*
Rch Dur	108.75 * ± 12.16*	163.00 * ± 14.06*	113.50 * ± 8.84*	128.42 * ± 7.46*	106.25 * ± 9.99*	130.75 * ± 8.08*	105.50 * ± 6.53*	114.17 * ± 4.96*
Attempts	0.70 * ± 0.11*	0.05 * ± 0.05*	0.00 * ± 0.00*	0.25 *± 0.06*	0.20 * ± 0.09*	0.05 * ± 0.05*	0.00 * ± 0.00*	0.08 * ± 0.04*

*The table summarizes the behavioral parameters before and after trimming. Estimated parameters: Whisker-T Poke: time lapse between first front wall whisker touch and nose poke. Nose-T Poke: time lapse between first front wall nose touch and nose poke. N. Nose T: number of nose touches preceding nose poking. Poke Reach-Start: time lapse between poke and start of reaching. Reach Duration: time lapse between paw lifting and paw crossing the slot. N. Attempts: a forelimb extension toward the slot without crossing it. In each box the top number is the mean value and the bottom number is the standard error, for *n* = 20. Note the significant increase in the number of nose touches after trimming (*p* = 0.000, Kruskal-Wallis test) while poke-start reach and reach duration do not change significantly (control vs. trimmed: *p* = 0.47 and 0.32, respectively, Kruskal-Wallis test)*.

**Table 2:** Behavioral parameters in control vs. infraorbital nerve (ION) severed rats.

	Control						ION 1–3D						ION 8–10D					
	R1	R2	R3	R4	R5	Group	R1	R2	R3	R4	R5	Group	R1	R2	R3	R4	R5	Group
Whisker-T Poke	***170.75*** *9.08*	***178.75*** *11.64*	***243.75*** *17.77*	***141.25*** *7.13*	***197.75*** *15.7*	***186.45*** *6.60*
Nose-T Poke							***641.25*** *104.44*	***752.5*** *138.81*	***675*** *159.86*	***752.75*** *108.41*	***781.25*** *112.87*	***720.55*** *55.75*	***413.00*** *79.34*	***602.75*** *107.66*	***386.25*** *82.42*	***248.50*** *59.76*	***257.50*** *50.92*	***381.6*** *36.76*
N-Nose T	***0.60*** *0.11*	***0.30*** *0.11*	***0.80*** *0.20*	***0.10*** *0.07*	***0.10*** *0.07*	***0.38*** *0.06*	***4.35*** *0.51*	***4.4*** *0.58*	***3.7*** *0.44*	***3.75*** *0.40*	***4*** *0.44*	***4.04*** *0.21*	***2.80*** *0.50*	***2.75*** *0.38*	***2.00*** *0.50*	***1.85*** *0.33*	***2.40*** *0.28*	***2.36*** *0.18*
Poke-End Reach	***258.10*** *6.19*	***391.14*** *18.29*	***393.00*** *14.05*	***307.75*** *6.13*	***325*** *10.3*	***330.08*** *10.76*	***795.00*** *127.44*	***767.62*** *56.03*	***771.82*** *103.67*	***400.50*** *56.66*	***713.03*** *56.33*	***519.47*** *46.54*	***557.73*** *58.07*	***298.81*** *13.20*	***300.65*** *41.05*	***338.41*** *17.42*	***325.00*** *30.76*	***342.63*** *15.93*
Attempts	***0.05*** *0.05*	***0.10*** *0.07*	***0.10*** *0.07*	***0*** *0*	***0*** *0*	***0.05*** *0.02*	***1.40*** *0.52*	***0.50*** *0.15*	***1.95*** *0.30*	***1.30*** *0.26*	***2.15*** *0.33*	***1.46*** *0.16*	***1.20*** *0.19*	***0.00*** *0*	***0.40*** *0.20*	***0.10*** *0.07*	***0.10*** *0.07*	***0.43*** *0.09*

*The table summarizes the behavioral parameters before and after ION severing at 1–3 and 8–10 days. Estimated parameters: Poke End Reach: time lapse between Poke and paw crossing the slot. Other parameters as in Table 1. In each box the top number is the mean value and the bottom number is the standard error, for *n* = 20. Note that at 1–3 days all behavioral parameters are significantly increased in comparison to controls (*p* = 0.000, Kruskal-Wallis test) while at 8–10 days only the poke-end reach values overlap values in controls (*p* = 0.000, Kruskal-Wallis test)*.

To analyze the Transport component under the different experimental conditions, we first, calculated the percentage of reaching-grasping movements with respect to the number of approaches. Interestingly, before the reappearance of successful reaching-grasping movement in ION-severed rats, they were seen repetitively touching the front wall with their forepaw in an attempt to locate the slot. To evaluate the degree to which nose poking affected the start and execution of reaching, we measured both the delay between the nose poke and reach start, and the duration of reaching. The reach start was defined by the frame in which the paw was first lifted off the box floor, and the end by the frame in which the paw crossed the slot. Usually in control rats the advance during reaching was a single continuous movement of the forelimb directed to the food to be grasped (Figures 1C,D); very rarely did the rats extend their forelimb towards the slot without crossing through movements (we defined as “attempts”). However, in ION-severed rats the reach start was not well identifiable in many trials, as reaching began with the paw neither leaning on the floor nor at rest. Hence, being unable to measure the nose poke–reach start delay and therefore the reach duration in this way, we instead measured the delay between nose poking and the end of reaching (see Tables 1, 2).

Finally, analysis of the Withdrawal relied on two precisely identifiable frames, specifically that in which the paw passed through the slot in the reaching-grasping movement, and that in which the paw re-crossed the slot during food retraction. The time interval between the two was used to calculate the duration of the reaching-grasping and withdrawal movements, i.e., the amount of time the paw spent beyond the slot.

### Statistical Analysis

All statistical analyses were performed using the R language and environment for statistical computing^2^. All data are represented in Table form as the mean ± standard error of *n* determinations, and using a bar chart to represent the mean values and the associated standard error. A binomial model was used to compute the probability of occurrence of the various behavioral movements in each experimental group. Pair-wise comparison of proportion was used to determine statistically significant differences in proportion between groups, using control values (control vs. ION-severed and control vs. trimmed) as the reference group. For behavioral parameters, a Kruskal-Wallis test by ranks, followed by Dunn’s *post hoc* test were used to determine statistically significant differences between experimental group values. When performing multiple comparisons, the Holm correction method for multiple hypotheses was used. The Pearson Chi-square goodness-of-fit test was applied to compare differences in movement frequency time-course after the whisker deafferentation. For all tests a significance level of *α* = 0.05 was set.

## Results

### Control Rat Behavior

Analysis of the video-recordings of the skilled reaching task at reduced speed not only provided an overview of their behavior in the box, but also enabled us to distinguish the three successive components of behavior that may potentially be influenced by whisker/nose input (Alaverdashvili and Whishaw, 2013), namely Orient, Transport and Withdrawal.

As mentioned above, the Orient component consisted of the rat walking from the back of the box, approaching the front wall, and ended with their locating the slot and poking their nose through to sniff the pellet. The walking was characterized by cyclical motion of the limbs and a head-down posture (Alaverdashvili et al., 2008) with exploratory whisking, during which only the longer mystacial macrovibrissae of row D and E contacted the wall (Berg and Kleinfeld, 2003). When the macrovibrissae contacted the front wall, the head was raised, the nose poked through the slot, and the rat located the food by sniffing (Whishaw and Tomie, 1988; Hermer-Vazquez et al., 2007; Figures 1A,B). In control rats (*n* = 8) the mean frequency of approaches to the front wall was 3.98 ± 0.28/min (range: 2.2–6.8/min). The ratio between approaching vs. poking was 100% in all control rats (*n* = 8), and the mean delay between whisker touch–nose poke, which measured the time the rat spent locating the slot, was 214.47 ± 6.75 ms (range: 100–530; *n* = 167; Figures 1A,B, single rat values in Tables 1, 2).

Once the rat located the food, the subsequent Transport response occurred, with the rat directing its forepaw through the slot towards the pellet. Analysis of the video-recordings at reduced speed confirmed that the reaching-grasping featured movement elements in the sequence described in the literature (Alaverdashvili et al., 2008; Alaverdashvili and Whishaw, 2013; Figure 1). Generally, the reaching movement started almost 193.96 ± 13.04 ms (range: 20–2015; *n* = 163) after the nose poke; it lasted for 138.16 ± 5.01 ms (range: 50–340; *n* = 160; Figures 1B–D, single rat values in Tables 1, 2) and was only rarely preceded by attempts (0.13 ± 0.03).

After grasping the food, the Withdrawal sequence was initiated, with the rat retracting its forepaw through the slot and directing the pellet to the mouth to eat. This behavior involved both forelimb and mouth movements (Figure 1E).

The surgery given to sham rats induced no effects on either the temporal sequence or the execution of the behavioral task (results not shown).

### Bilaterally Trimmed Rat Behavior

After macrovibrissae trimming, the mean frequency of approaches to the front wall was not significantly different with respect to controls (trimmed vs. control: 3.98 ± 0.28/min vs. 3.28 ± 0.23 min, *n* = 3 *p* = 0.45; Kruskal-Wallis test by ranks), and, like control rats, the ratio between trimmed rats’ poking vs. approaching was 100%. That being said, there was a change in the way the trimmed rats detected the front wall and located the slot. Indeed before trimming, the same rats detected the front wall by whisker touch, the snout not touching it before the nose poke (Figures 2A,B vs. Figure 1A), while after trimming they detected the presence of the wall by hitting it with the nose, only locating the slot after repeated nose touches. Unsurprisingly, therefore, the number of nose touches preceding poking was significantly higher in trimmed rats than in controls (mean number of touches in trimmed vs. control: 2.45 ± 0.14 vs. 0.52 ± 0.07, *p* = 0.0000; Wilcoxon rank sum test, single rat values in Table 1). The mean delay between first nose touch and nose poke was 202.74 ± 16.19 ms (range: 30–675; *n* = 61; Figures 2A–C, single rat values in Table 1), and in each trial the mean delay between the first nose touch and nose poke was directly related to the number of touches (*R* = 0.79; *p* = 0.000; Pearson’s product moment correlation coefficient). This delay measured the time spent locating the slot by nose touches, and was not comparable to the corresponding whisker touch–nose poke interval in the same rats before trimming (see Table 1). In contrast, macrovibrissae trimming resulted in no changes in the reaching-grasping-retracting components of skilled reaching: neither the number nor the temporal sequence of the task differed with respect to the same rat before trimming (Figures 2D–F). In particular, there was no difference between either the nose poke–reach start delay or the reach duration after trimming when compared to controls (Table 1 trimmed vs. control: *p* = 0.47 and *p* = 0.32, respectively; Wilcoxon rank sum test).

### Bilaterally ION-severed Rat Behavior

#### Qualitative Behavior Description

**Figure 3:**
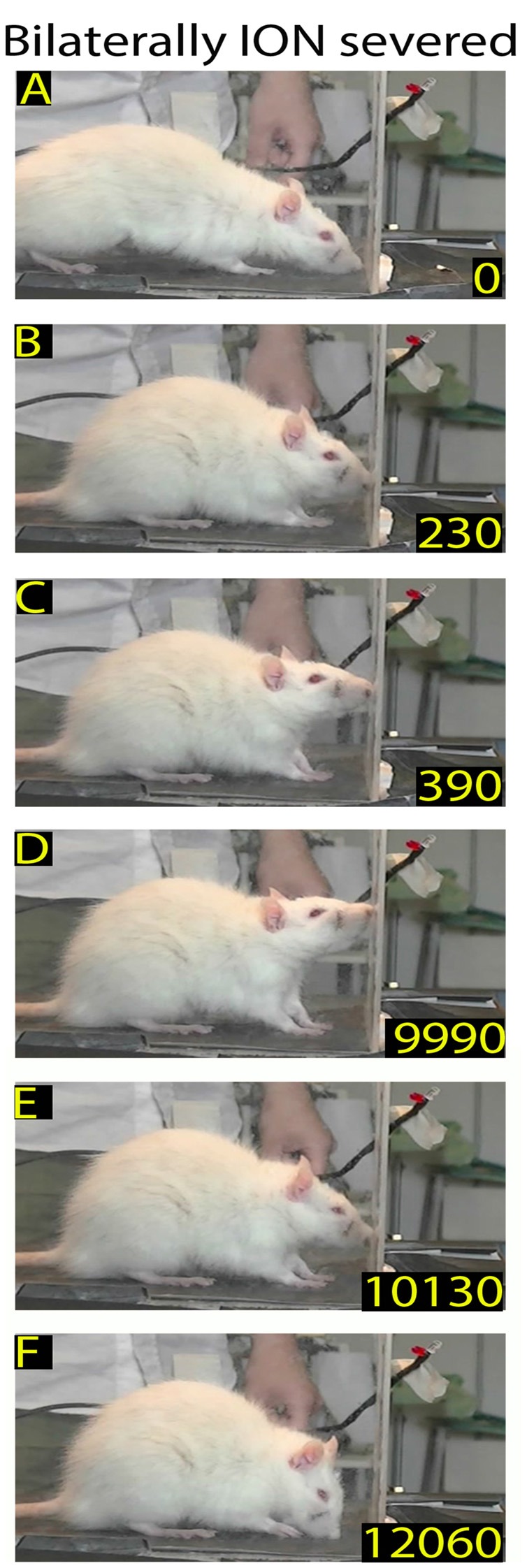
Example of rat behavior after bilateral infraorbital nerve (ION) severing: video-recording from Rat 2 4 days after surgery. The rat approaches the front wall **(A)**, does not insert its nose into the slot, stands in place **(B–E)**, and then moves away **(F)**. Note that in this interrupted sequence the rat stays for a long time in front of the wall.

**Figure 4:**
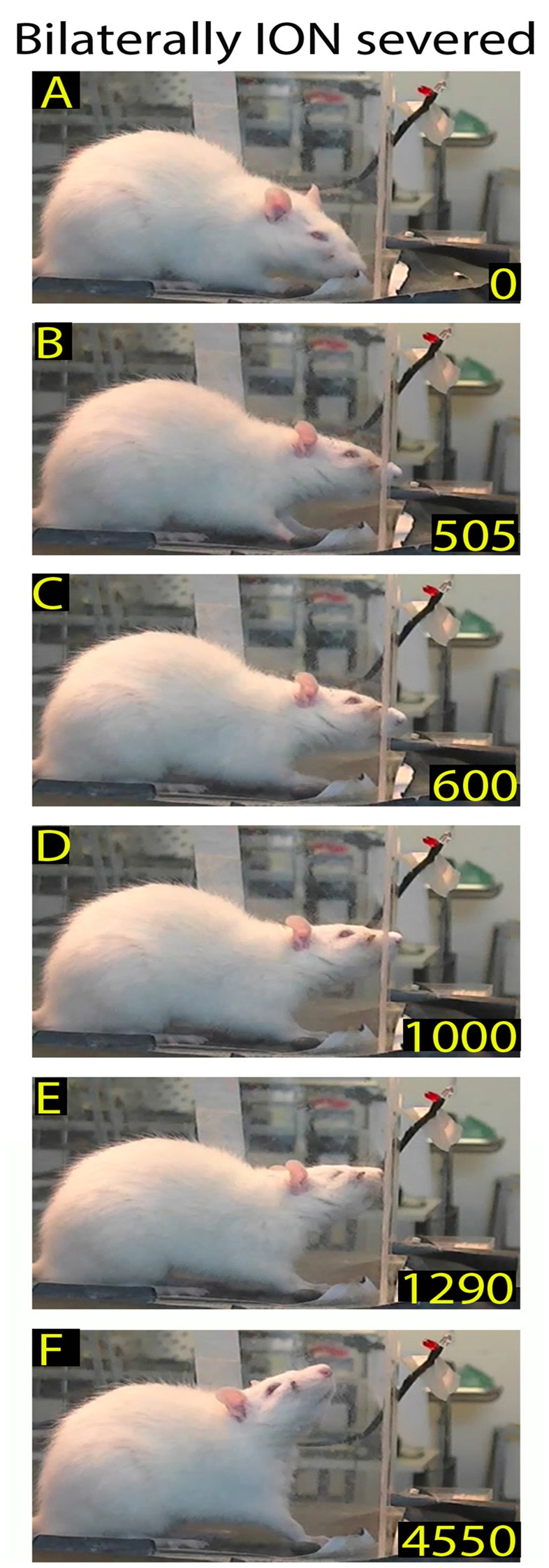
Example of rat behavior after bilateral ION severing: video-recording from Rat 2 10 days after surgery. The rat approaches the front wall **(A)**, inserts the nose into the slot **(B)**, and then, after standing there for a long time **(C–E)**, moves away **(F)**. Note that although the insertion of the nose into the slot is prolonged, the rat does not execute the reaching-grasping-retracting movement.

**Figure 5:**
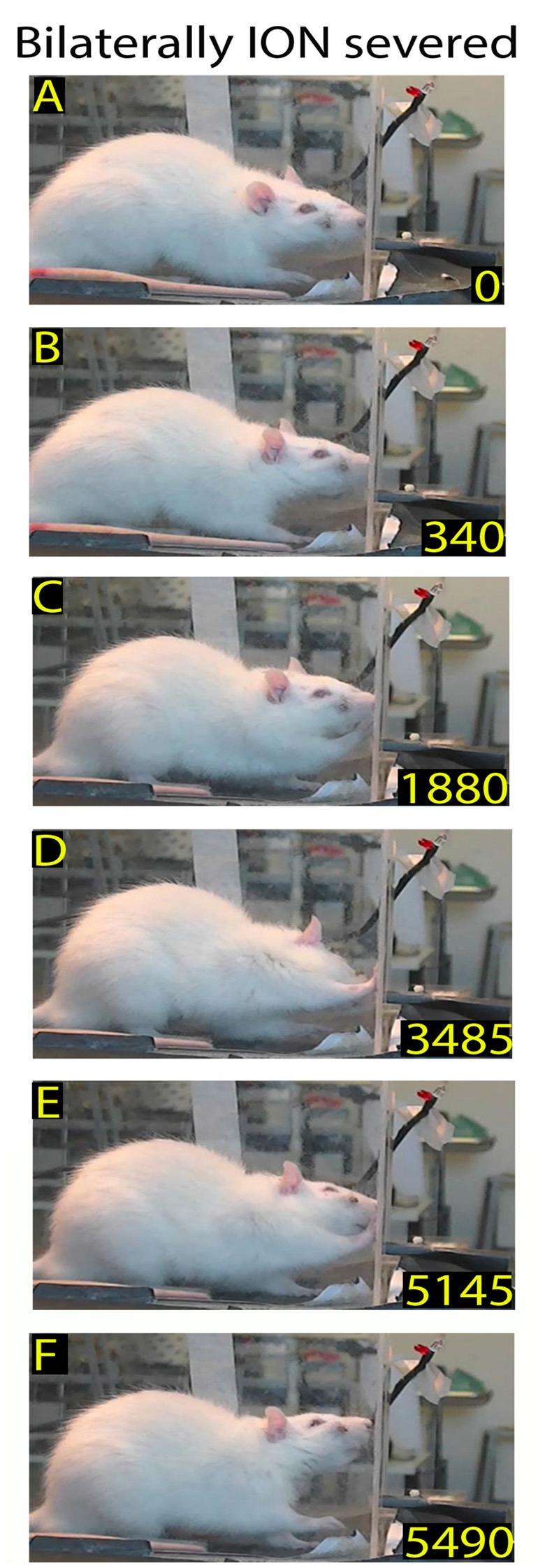
Example of rat behavior after bilateral ION severing: video-recording from Rat 2 12 days after surgery. The rat approaches the front wall **(A)** and explores it with repetitive forelimb movements **(B–E)** before moving away **(F)**. Note that, despite the prolonged exploration of the front wall, the rat does not insert its nose into the slot or execute the reaching-grasping-retracting movement.

**Figure 6:**
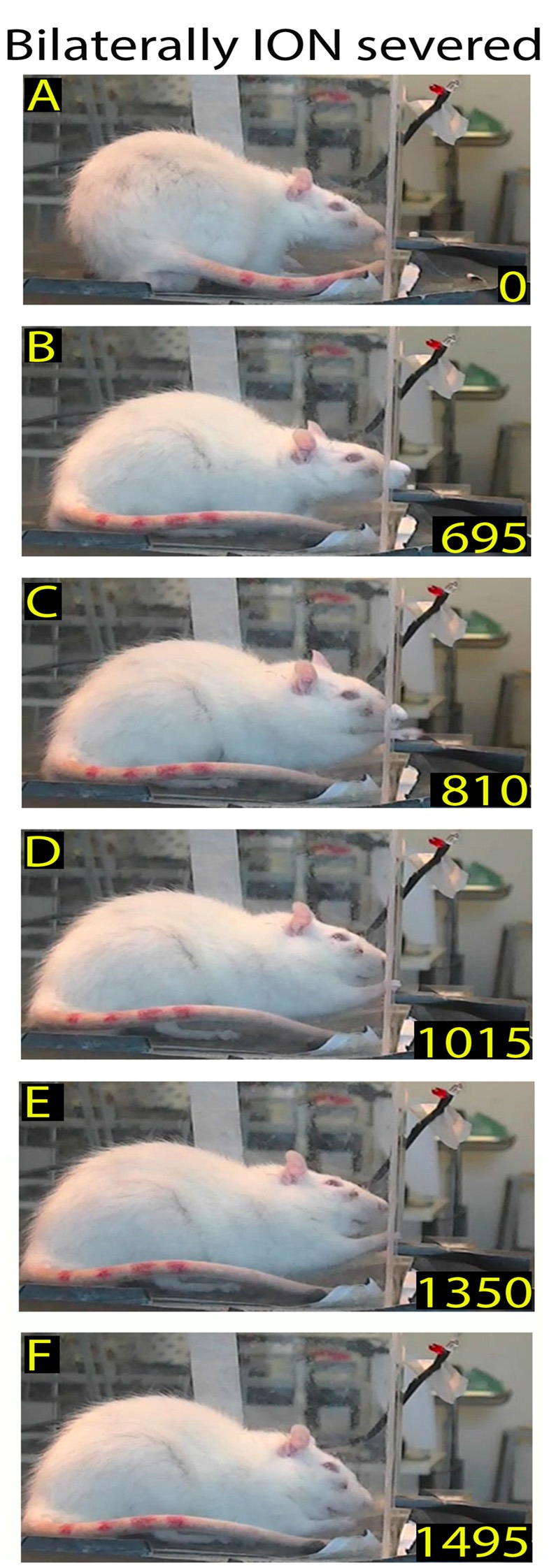
Example of restored reaching-grasping and retracting movements in bilaterally ION-severed rat: video-recording from Rat 2 12 days after surgery. The rat approaches the front wall **(A)**, inserts its nose into the slot **(B)**, and slowly executes the reach-grasp-retract sequence **(C–F)**. Note that this is one of the first complete trials performed by the rat after the lesion, and that the sequence is performed more slowly with respect to the trial in Figure 1.

**Figure 7:**
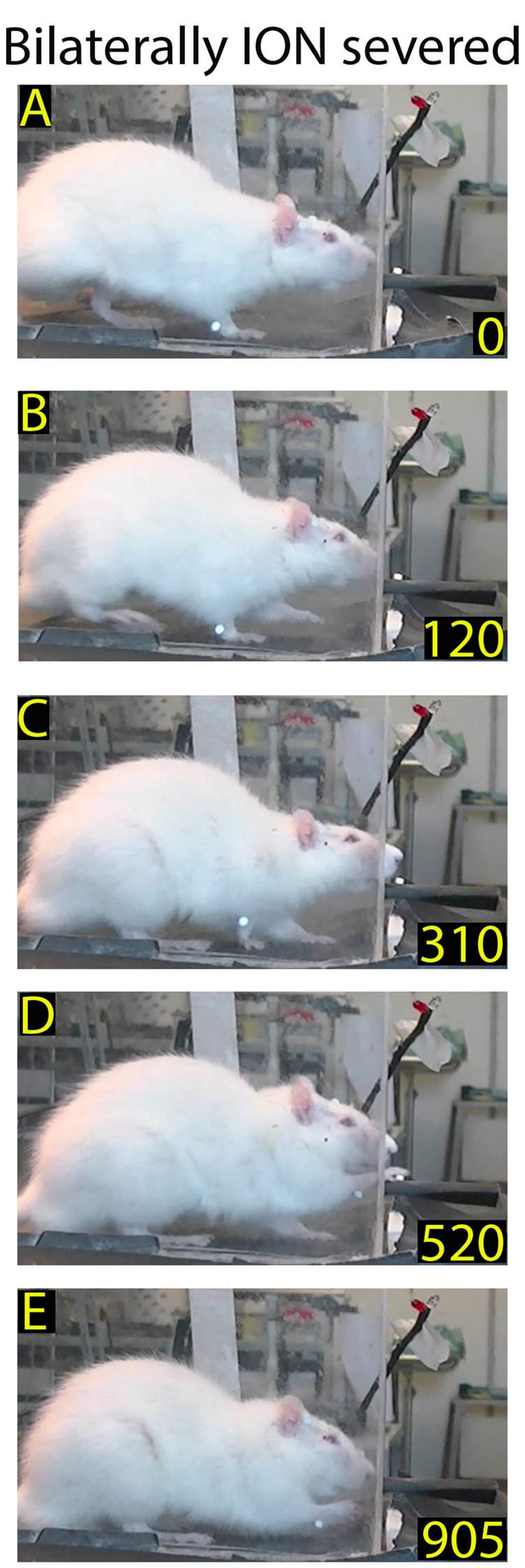
Example of normalized reaching-grasping and retracting movements in bilaterally ION-severed rat: video-recording from Rat 2 17 days after surgery. Note that the nose contacts with the front wall during approaching are abnormal **(A,B)** while reaching/grasping and retract timing are similar to those shown in Figure 1. **(C–E)** Markers are present (see “Materials and Methods” section).

In rats with bilateral ION-severing (*n* = 5), whisker deafferentation induced a marked effect on the structure of the skilled behavior. Video-recordings were executed daily until about 3 weeks, when the rat recovered the reaching-grasping movement, but changes were already detectable in the first post-surgical recording sessions. Specifically, in 67% of trials (*n* = 318), the ION-severed rat, after arriving at the front wall of the box, stopped in front of the slot, but failed to locate it and insert its nose; after standing in front of the wall, the rat went back to the rear of the box. In other words, the behavioral sequence of the task was interrupted before the end of Orient (Figure 3). In the remaining 33% of trials, the ION-severed rat, when arriving at the front wall of the box, inserted its nose through the slot and sniffed but without starting the reaching movement; while sniffing, the preferred forelimb remained resting on the floor of the box (Figure 4). In this case, the behavioral sequence was considered to interrupt before Transport. From the 6th day onward, in some trials the animal, approaching the front wall, explored it with repetitive forelimb movements for a few seconds, but without inserting its nose into the slot or executing a reaching movement to grasp the pellet (Figure 5). The reaching-grasping movement reappeared on either the same day or the subsequent day, and was then followed by Withdrawal (Figure 6). The full reaching-grasping-retracting sequence reappeared between 6 days and 12 days after ION severing, depending on the animal. Frame-by-frame video analysis showed that, although the complete Transport and Withdrawal sequence of behavior was restored, it initially had a longer duration than in controls (Figure 6). After about 3 weeks, however, the rat executed the task in a time comparable to that of controls (Figure 7).

#### Quantitative Behavior Description

**Figure 8:**
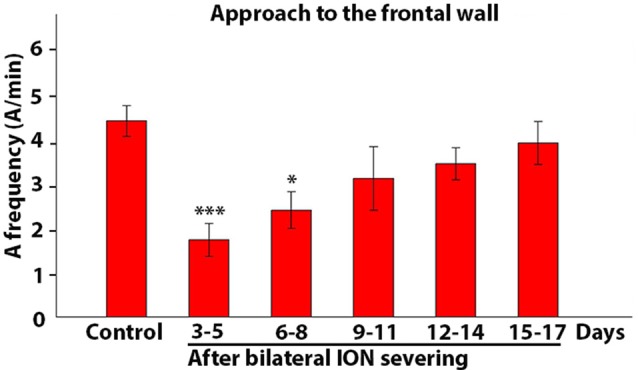
Frequency of approach to the front wall. The histogram presents data on five rats collected in control conditions and at different time intervals after bilateral ION severing. Each bar shows the mean and standard error of approaches expressed as a frequency (A/min). Note that the frequency is significantly reduced with respect to control at 3–5 and 6–8 days (***P = 0.001; *P = 0.038, *post hoc* Dunn’s test).

The observations obtained through qualitative analysis were then analyzed quantitatively in order to test whether and how ION-mediated information plays a role in guiding and triggering one or more components of the movement sequence involved in rat skilled reaching behavior. Hence, in ION-severed rats, we first analyzed the mean frequency of approaches to the front wall. Kruskal-Wallis test comparing control and ION-severed rat revealed a major significant effect for bilateral ION-severing (Kruskal-Wallis *chi-squared* = 21.84; *P* = 0.0000). As regards the approach frequency, Holm *post hoc* testing showed that this was significantly reduced at days 3–5 and 6–8 with respect to the same rats before ION severing (3–5 days: *P* = 0.001; *n* = 12 sessions with 318 trials; 6–8 days: *P* = 0.038; *n* = 10 sessions with 650 trials), but from days 9 to 11 onwards, the frequency did not appear significantly different from controls (*P* = 0.87; *n* = 5 with 365 trials; Figure 8).

**Figure 9:**
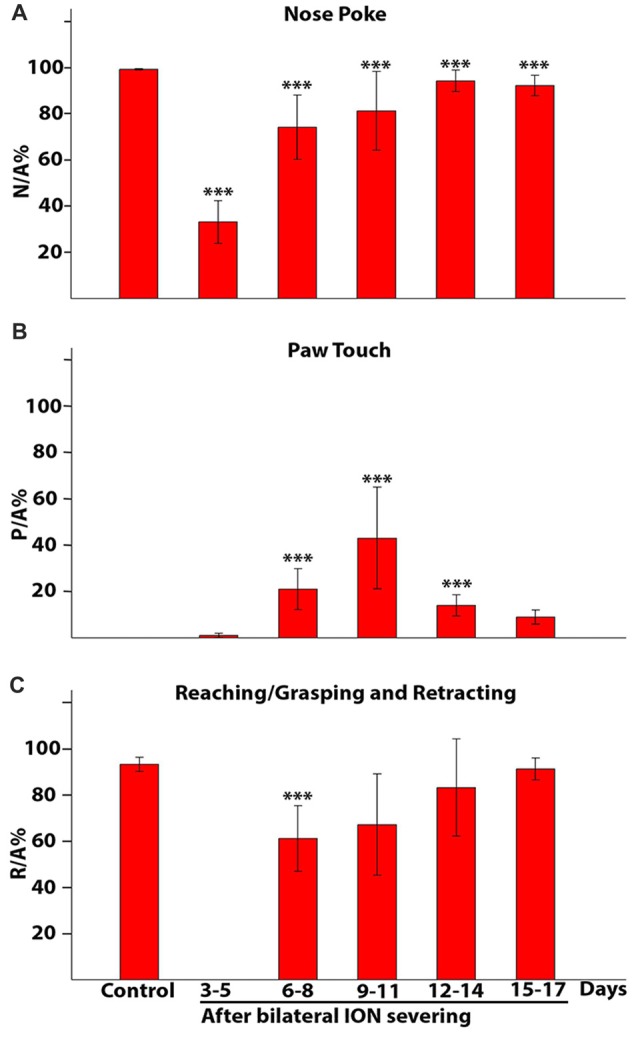
Percentage of nose poke **(A)**, paw touch **(B)** and reaching/grasping **(C)** expressed as a ratio with respect to the approaches. The histograms present data from five rats collected in control conditions and at different time intervals after bilateral ION severing. Each bar shows the mean and standard error. **(A)** Nose poke percentage expressed as a ratio with respect to the approach (N/A%). Note that in controls all approaches to the front wall are followed by insertion of the nose into the slot (100%), whereas 3–5 days after surgery, in about 70% of the approaches the rat fails to insert its nose into the slot and in subsequent days percentages are still significantly lower than in controls (***P = 0.000, pair-wise comparison of proportion). **(B)** Paw touch percentage expressed as a ratio with respect to the approach (P/A%). Note that paw touch is not present in controls and showed the greatest increase over 9–11 days (3–5 days vs. other groups: ***P = 0.000, pair-wise comparison of proportion). **(C)** Reaching/grasping percentage expressed as a ratio with respect to the approach (R/A%). Note that movements are totally absent 3–5 days after the lesion, reappear in the following days and at 6–8 days are still significantly different from control (***P = 0.000, pair-wise comparison of proportion).

Figures 9A–C showed how the movement components changed over time in ION-severed rats with respect to controls. Each bar represented the percentage ratio between the number of times a given event occurred and the number of approaches in a specific time interval. For example, Figure 9A showed how many times the animals inserted their nose into the slot with respect to the number of approaches. Before injury, all approaches to the front wall were followed by nose insertion into the slot. However, 3–5 days after surgery, pair-wise comparison of proportions revealed a highly significant effect of ION severing on the percentage of nose insertion into the slot (*P* = 0.000; *n* = 12 sessions with 318 trials). In subsequent days percentages are still significantly lower than in controls (***P = 0.000; *n* = 28 sessions with 1845 trials; pair-wise comparison of proportion). Likewise, Figure 9B showed how many times the animal, approaching the front wall, explored it with repetitive paw movements. Pair-wise comparison of proportions comparing control and ION-severed rats showed that bilateral ION severing had a major significant effect in this regard (*P* = 0.0018; *n* = 12 sessions with 318 trials). Indeed, this behavior was not present in controls, appeared at very low frequency 3–5 days post-injury, and showed a significant increase at 6–8, 9–11 and 12–14 days (*P* = 0.0000; *n* = 27 sessions with 1842 trials, pair-wise comparison of proportions computed using a binomial model). At 15–17 days, paw touching was still present, but its percentage was not significantly different from 3 days to 5 days (*P* = 0.23; *n* = 13 sessions with 1077 trials, pair-wise comparison of proportions computed using a binomial model). Similarly, Figure 9C showed how many times the animal executed the reaching-grasping-retracting sequence with respect to the number of approaches. Once again, pair-wise comparison of proportions comparing control and ION-severed rats showed a major significant effect, as the movement was totally absent 3–5 days after lesion (*P* = 0.0000; *n* = 12 sessions with 318 trials), but at 6–8 days its frequency was significantly different (*P* = 0.0000; *n* = 10 sessions with 650 trials; pair-wise comparison of proportions computed using a binomial model). On subsequent days, however, this movement frequency was not significantly different to controls (9–11: *P* = 0.20; 12–14:* P* = 0.72; 15–17:* P* = 0.74; pair-wise comparison of proportions computed using a binomial model).

The population data from the five animals tells us nothing about when the full reaching-grasping-retracting sequence reappeared in each rat; this is shown in Table 3, in which the ratio between forelimb movements and approaches was expressed as a percentage value for each animal. In this Table, 0 means that no approach was followed by reaching-grasping, while 1 indicates that each approach was followed by reaching-grasping. As evident from the Table 3, in three rats (R3, R4 and R5) reaching-grasping reappeared 6–8 days after lesion, returning to baseline levels in 9–11 days. In contrast, reaching-grasping reappeared later in R1 and R2, and R1 failed to recover baseline performance over 17th days of recordings (R2, R3, R4 and R5 vs. R1: *P* = 0.0001, *χ*^2^ test, 2X2 contingency table).

**Table 3:** Reaching/grasping frequency in relation to approach.

	Control	3–5 days	6–8 days	9–11 days	12–14 days	15–17 days
R1	0.82	0	0	0.35	0.34	0.63
R2	0.93	0	0	0	0.76	0.99
R3	0.99	0	0.75	1	1	0.99
R4	0.99	0	0.98	1	1	1
R5	1	0	0.88	1	1	0.99
Average	0.95	0	0.52	0.67	0.82	0.92
Std error	0.03	0	0.22	0.21	0.13	0.07

*Ratio between reaching/grasping and approach expressed as a percentage for each animal in controls and at different time intervals after ION severing. Note that in rats R3, R4 and R5 the movement reappears 6–8 days after the lesion, while in R1 and R2 the movement reappears later*.

We also considered the ratio between reaching-grasping and nose poke, expressed as a percentage value for each animal; this measured how many times each rat performed the reaching-grasping movement after inserting its nose into the slot, and gave an indication of the triggering power of nose insertion for the subsequent reaching-grasping movement. As shown in Table 4, all control rats presented a close relationship (almost 1) between reaching-grasping and nose poke. At 3–5 days after surgery, on the other hand, nose poke did not trigger reaching-grasping in any rats (0); in three rats (R3, R4 and R5) nose poke began to trigger reaching-grasping once again after 6–8 days, while in the other two rats (R1 and R2) this trigger function reappeared later (R3, R4 and R5 vs. R4 and R5: *P* = 0.0001, *χ*^2^, 2X2 contingency table).

**Table 4:** Reaching/grasping frequency in relation to poke.

	Control	3–5 days	6–8 days	9–11 days	12–14 days	15–17 days
R1	0.88	0	0	0.5	0.52	0.90
R2	0.93	0	0	0	0.8	0.99
R3	1	0	0.8	1	1	1
R4	1	0	1	1	1	1
R5	1	0	0.82	1	1	0.99
Average	0.96	0	0.45	0.70	0.86	0.98
Std error	0.02	0	0.24	0.22	0.09	0.02

*Ratio between reaching/grasping and poke expressed as a percentage for each animal in controls and at different time intervals after ION severing. This ratio gives an indication of the triggering power of nose insertion into the slot for the subsequent reaching/grasping movement. Note that all control rats present a ratio of almost 1:1. At 3–5 days, poke does not trigger reaching/grasping; in R3, R4 and R5, poke triggers reaching/grasping after 6–8 days, and in R1 and R2 this triggering function reappears later*.

To quantify each component of the skilled reaching task, both at the time of its reappearance and in the following days, we analyzed trials obtained in control rats, and in the same rats after bilateral ION severing 1–3 and 8–10 days after the reappearance of reaching behavior. Like trimmed rats, ION-severed rats detected the presence of the wall by hitting it with their nose, and then located the slot through repetitive nose touches. A clear increase in the mean number of nose touches was evident at 1–3 days (*P* = 0.0000; *n* trials = 100; Kruskal-Wallis rank sum test; Table 2) and at 8–10 days the values decreased but were still significantly higher in comparison to controls (*P* = 0.0003; *n* trials = 100; Kruskal-Wallis rank sum test; Table 2).

As for the reach start, in control rats it was easily identified when the paw lifted from the box floor; conversely, in ION-severed rats the reach often started after several forelimb attempts with the paw in a raised position. Hence, we decided to define the delay between the nose poke and the end of reach as an indirect measure of reach duration. A clear increase in mean poke–reach end delay was evident at 1–3 days (*P* = 0.0000; *n* trials = 104; Kruskal-Wallis rank sum test; Table 2), and at 8–10 days the values overlapped those of controls (*P* = 0.25; *n* trials = 99; Kruskal-Wallis rank sum test; Table 2). Otherwise, the attempts increased 1–3 days (*P* = 0.0000; *n* trials = 100; Kruskal-Wallis rank sum test; Table 2), and at 8–10 days the values were still significantly higher in comparison to controls (*P* = 0.04; *n* trials = 100; Kruskal-Wallis rank sum test; Table 2).

**Figure 10:**
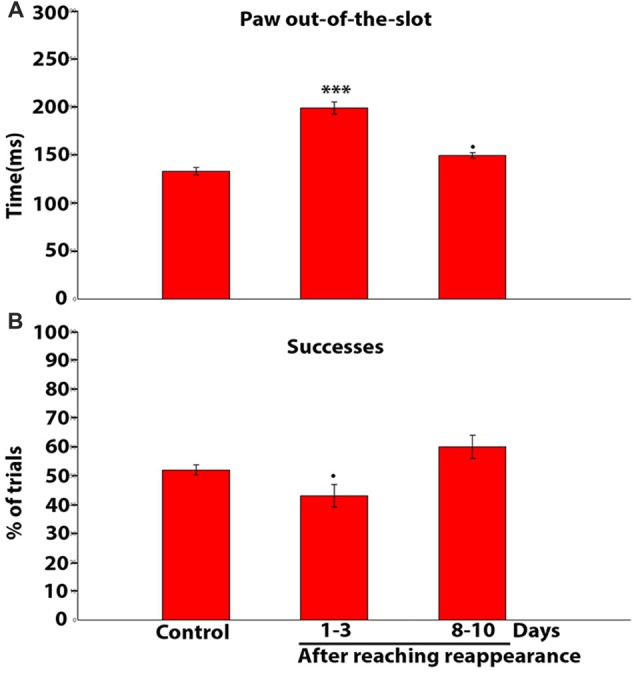
**(A)** Population data histogram showing the average time spent by the paw beyond the slot during reaching-grasping-retracting movement. Each bar shows the mean and standard error in ms. Note that the time is significantly increased with respect to control at 1–3 days (***P = 0.0001, chi square test); and is slightly increased on days 8–10 (^•^*P* = 0.0523, chi square test). **(B)** Population data histogram of the success percentage in controls and after reaching reappearance in ION severed rats. Note that the success percentage is slightly reduced only on days 1–3 after reaching reappearance (^•^*P* = 0.0952, chi square test).

We then set out to quantify the time the paw spent beyond the slot during the reaching-grasping-retracting movement in each trial. Accordingly, Figure 10A, a mean population data plot, shows the mean values of these measures obtained in control rats, and in bilaterally ION-severed rats 1–3 and 8–10 days after the reappearance of reaching behavior. A significant increase in mean duration is evident at 1–3 days (*P* = 0.0000; *n* trials = 104; Kruskal-Wallis rank sum test), and at 8–10 days the values were still increased, tending towards significance, in comparison to controls (*P* = 0.0523; *n* trials = 99; Kruskal-Wallis rank sum test). Finally, we investigated whether this slowed execution of the reach-grasp-retract sequence corresponded to a reduced ability to grasp pellets successfully. As shown in the histogram in Figure 10B, successes were slightly reduced only 1–3 days after the reappearance of reaching with a trend towards significance (*P* = 0.0952; *n* trials = 657; chi square test), in comparison to control.

## Discussion

This experiment was designed to investigate whether and how rats use their ION-mediated whisker/nose sense in skilled motor behavior. Indeed, previous studies have found a significant role of whisker sense in many types of motor behavior (Anjum et al., 2006; Hartmann, 2011; Grant et al., 2012), but its role in skilled reaching has never previously been explored. Skilled reaching is a composite behavior (Alaverdashvili et al., 2008), and, in order to better identify the specific roles of whisker/nose sense in this pattern, we considered each trial as composed of three behavioral responses: Orient (approach, slot location and nose poke), Transport (reaching-grasping) and Withdrawal (pellet retraction; Alaverdashvili and Whishaw, 2013).

As regards Orient, it has been suggested that the macro and microvibrissae are often employed together in this behavioral task: the macrovibrissae sample spatially in order to direct the microvibrissae (Brecht et al., 1997; Hartmann, 2011; Grant et al., 2012). Once it has approached the front wall, the rat switches from using its whiskers to explore the floor surface (Arkley et al., 2014) to using them to explore the front wall in order to determine the position of the slot and inserting the snout. In our experiment, control rats freely approached the front wall and contacted it with their macrovibrissae, while the microvibrissae came into contact with the wall during poking behavior. According to this sequence, it seems that macrovibrissae sampled the presence of the front wall and the location of the slot while microvibrissae came into play during poking, and played a permissive role in the start of reach. Indeed, after macrovibrissae trimming, the rat’s explorative capacity inside the box seemed unaltered when evaluated as the number of approaches, while its behavior in relation to the front wall differed substantially. Specifically, the rat appeared to explore the front wall as an unexpected object (Prescott et al., 2011; Grant et al., 2012), only detecting its presence by hitting it with its nose, and then located the slot by repetitive nose touches. Nonetheless, it is clear from our results that macrovibrissae trimming did not affect the subsequent reaching-grasping-retracting components of skilled reaching.

In contrast, bilaterally ION-severed rats strongly and persistently displayed a reduced ability to explore the box and detect the front wall. These rats not only performed a smaller number of approaches to the front wall, but also showed a transient but significant reduction in nose-poke frequency, and a persistent, strongly significant increase in nose touches. Moreover, ION-severed rats showed an increase in the nose touch–poke delay to a greater extent than trimmed rats. This suggests that subtraction of whisker/nose sense strongly and persistently impairs the ability of the rat to explore the front wall and locate the slot. Interestingly, after losing its whisker/nose sense, the rat recovered the ability to locate the pellet using its sense of smell, although less efficiently. Though data from experiments in which olfaction is suppressed and whisker/nose sense preserved indicate that olfaction is used to locate food and direct reaching (Whishaw and Tomie, 1989; Hermer-Vazquez et al., 2007). We observed that when the bilaterally ION-severed rat succeeded in placing its nose in the slot, the reaching-grasping movement does not automatically follow. Having observed that macrovibrissae trimming does not alter the behavioral sequence recorded in controls, it follows therefore that microvibrissae/nose mediated sensory information may be the trigger for the reaching movement in rats. Indeed, though before bilateral ION severing all rats explored the front wall of the box exclusively with their whiskers—with the forelimbs being involved in postural adjustments (Alaverdashvili et al., 2008)—from the 6th day after lesion, in some trials rats explored the front wall with repetitive forelimb movements for a few seconds without inserting their nose into the slot or reaching to grasp the pellet. We therefore hypothesize that these forelimb movements could be the expression of a sensorimotor strategy utilized to collect sensory information from active paw sense to compensate for the absence of whisker/nose sense.

Since somatosensory input is associated with grasping, forelimb retraction and placing the food into the mouth (Sacrey and Whishaw, 2012; Karl et al., 2013). We hypothesized that reaching-grasping-retracting may be affected by suppression of whisker/nose sense. Hence, to obtain an estimate of the duration of the reaching-grasping-retracting, we calculated the time the paw spent beyond the slot. As expected, our data show that this interval increased significantly after lesion; in contrast, success frequency immediately after the reappearance of reaching (1–3 days) was only slightly reduced.

In conclusion the present results show that trimming and ION severing clearly induce different behavioral deficits. In control rat the front wall localization occurs with the whiskers positioned at the maximum protraction for a sequence of two-three whisks. The front wall localization occurs with initial macrovibrissal contact and microvibrissal/nose contact in the final sequence during poking. The macrovibrissae contact with the front wall guides the head movement towards the slot. Specifically, macrovibrissae detect the distance of the front wall and the position and shape of the slot in order to insert the nose into it. After macrovibrissae trimming the rat detects the position of both front wall and slot by means of repeated nose/microvibrissae touches that guide the head movement. After ION severing, the combined loss of whiskers and nose signals alters all steps of the trial and interrupts the sequence of the task. During the whole task execution the rat behaves in a way compatible with the loss of peripersonal space perception.

A major aspect of our findings is that post-lesion behavioral deficits occur immediately after injury, and are consistent in all bilaterally ION-severed rats. Interestingly, however, bilaterally ION-severed rats present different recovery times for reaching-grasping and retracting movements. Although we cannot provide a direct explanation for these different recovery rates, we did note that rats with slower recovery spent more time achieving consistent performance in the skilled reaching task before ION severing (almost 6–7 vs. 4–5 weeks).

At this point the significant question arises as to whether functional recovery is a peripheral or central phenomenon. Our data strongly support the latter hypothesis, since whisker-pad sense was shown to be blocked throughout the recording period, and all rats presented a severe and persistent alteration in spontaneous grasping, with no recovery. At the end of recording sessions, we observe the rat spontaneously grasping pellets dropped on the box floor as a final reward. While both control and trimmed rat normally grasps the pellet on the floor with their mouth, the bilaterally ION-severed rat instead grasps the pellet with its paw when the pellet fortuitously get in touch with the paw.

The last question arising from our data regards how and where the recovery mechanisms take place. It is known that rats’ olfactory, whisker/nose sense and proprioceptive input interact with one another to guide skilled reaching behavior (Whishaw and Tomie, 1989; Kleinfeld et al., 2014), and that projections from various cortical areas, including somatosensory, visual, auditory and entorhinal cortices, converge on the posterior parietal cortex (Reep et al., 1994; Lee et al., 2011; Bonnevie et al., 2013), which plays a causal role in guiding sensorimotor responses (Save et al., 2005; Raposo et al., 2014; Licata et al., 2017). In conclusion, therefore, we suggest that permanent whisker/nose sense removal may have far-reaching effects in all connected central sensorimotor structures normally used by rat in this behavior, and that compensation could be mediated by olfactory information from the entorhinal cortex converging on the posterior parietal area.

## Author Contributions

GF and PP designed the experiment. GF, CL and PP performed the experiment and collected the data. CL and PP analyzed the data. GF and CL wrote the manuscript.

## Conflict of Interest Statement

The authors declare that the research was conducted in the absence of any commercial or financial relationships that could be construed as a potential conflict of interest.

## Footnotes

^1^www.avidemux.org

^2^www.R-project.org/
